# Medical Applications at CERN and the ENLIGHT Network

**DOI:** 10.3389/fonc.2016.00009

**Published:** 2016-01-25

**Authors:** Manjit Dosanjh, Manuela Cirilli, Steve Myers, Sparsh Navin

**Affiliations:** ^1^CERN, Geneva, Switzerland

**Keywords:** particle physics, imaging, radiotherapy, detectors, accelerators, hadron therapy

## Abstract

State-of-the-art techniques derived from particle accelerators, detectors, and physics computing are routinely used in clinical practice and medical research centers: from imaging technologies to dedicated accelerators for cancer therapy and nuclear medicine, simulations, and data analytics. Principles of particle physics themselves are the foundation of a cutting edge radiotherapy technique for cancer treatment: hadron therapy. This article is an overview of the involvement of CERN, the European Organization for Nuclear Research, in medical applications, with specific focus on hadron therapy. It also presents the history, achievements, and future scientific goals of the European Network for Light Ion Hadron Therapy, whose co-ordination office is at CERN.

## Introduction

Physics underpins many techniques and technologies that are used for both diagnosis and treatment of a variety of diseases: discoveries from basic physics research have been closely linked to medicine for centuries, and numerous tools developed by physicists to pursue their scientific goals have found their way into hospitals around the world.

In particular, innovative ideas and technologies originating from particle physics have been playing an increasingly important role in medicine over the last 100 years since the advent of radiation-based medical diagnosis and treatment. Nowadays, state-of-the-art techniques derived from particle accelerators, detectors, and physics computing are routinely used in clinical practice and medical research centers: from technology for Positron Emission Tomography (PET) scanners, to dedicated accelerators for cancer therapy, simulations, and data analytics.

Hadron therapy, also known as particle therapy, epitomizes the connection between basic physics (the property of particles traversing matter) and medicine (cancer treatment). Using protons and other ions to treat cancer demands large accelerators; this links radiation therapy to the development of increasingly powerful accelerators for particle physics research. At the same time, hadron therapy is a truly multidisciplinary venture that requires input from oncologists, radiation biologists, medical physicists, particle physicists, and computing scientists.

**Figure 1:**
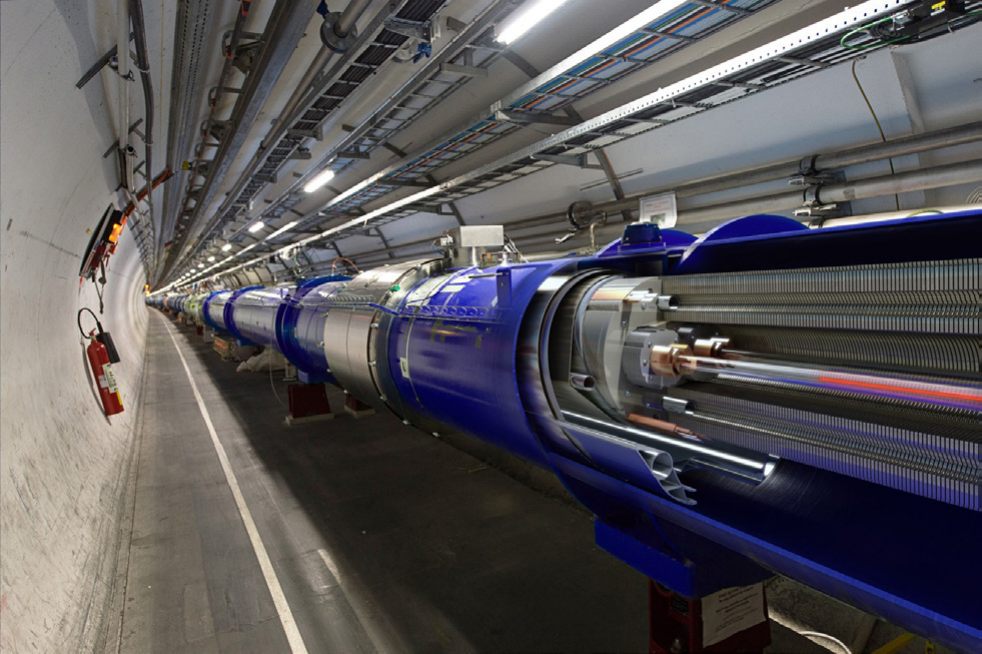
**The Large Hadron Collider (LHC)**. CERN’s flagship project, the LHC is a 27-km circular accelerator where protons collide at a center-of-mass energy of 13 TeV. The initial 3-year LHC run, which began with a collision energy of 7 TeV, rising to 8 TeV, led to the discovery of the Higgs boson in 2012.

CERN, the European Organization for Nuclear Research (Figure 1) is the world’s largest particle physics laboratory. Its contribution to particle physics research and related technologies has been outstanding since its establishment in 1954. CERN’s primary mission is basic research in particle physics; yet, the laboratory seeks possibilities to transfer its know-how and technology to other fields, including health, in order to maximize the societal impact of its research.

In this context, the European Network for Light Ion Hadron Therapy (ENLIGHT) was launched at CERN in 2002, to connect research centers, institutions, and scientists, involved in the research, promotion, and realization of hadron therapy in Europe.

This article briefly recaps the history of medical applications at CERN and then provides an overview of the present situation, with particular emphasis on research and development connected to hadron therapy. The role of ENLIGHT and its research program are also covered.

## From Particle Physics to Health

Fundamental research in particle physics not only pushes back the boundaries of our knowledge of the Universe but also catalyzes innovative technology developments: frontier instruments like the Large Hadron Collider (LHC) and its detectors require technologies and performance that exceed the available industrial know-how.

The three technology pillars of particle physics – accelerators, detectors, and computing tools – have all found their way into the medical field. Accelerators have been used for radiation therapy of cancer for decades. Medical applications of particle detectors are epitomized by PET, which is a direct application of light-sensing techniques. Data handling and simulation tools developed by physicists have found use in the biomedical field, for example, in establishing personalized treatment plans.

A host of highly specialized technologies is associated with each of the three pillars. To transfer this wealth of know-how to medicine, it is essential not only to identify which technologies are potentially interesting for medical applications but also what is their relevance for the medical community. In order to maximize the societal benefit from particle physics, the physicists’ and medical doctors’ research activities should be harmonized. This can only be achieved through multidisciplinary networks and collaborations, where scientists of different specialties all make their contributions to establish a common roadmap. Physicists, engineers, and computer scientists would share their knowledge and technologies, thus giving first-hand information to the medical community on the latest technical progress; conversely, doctors and biologists would present their needs and vision for the medical tools of the future, thus triggering breakthrough ideas and technical developments in specific areas.

Experience gained in particle and accelerator physics may serve more than just a technology, which shapes up the medicine of the future. Scientific collaborations in particle physics have been bringing together thousands of scientists from every corner of the world to work on the largest and most complex experiments ever conducted by mankind. Collaboration has become second nature of particle physicists, who have learned to work collectively on a common goal and who rely on mutual consensus to make decisions. The collaborative model of particle physics, represented in literature (1) has been translated into a flexible yet effective management structure for the experiments. This new paradigm for teamwork has proven its worth, and could serve as a model to follow for the emerging multidisciplinary ventures in medical applications.

### CERN and Medical Applications: A Brief History

Over the past 60 years, CERN has developed a world-renowned expertise in the three core technology domains of particle physics – accelerators, detectors, and large-scale computing – as well as in many ancillary technologies. The transfer of technology and know-how has always been one of the missions of the Laboratory, even before the formal establishment of a technology transfer unit in the late 80s. Early medical applications activities date back to the 1970s and have been initiated mostly by individual interests.

The multi-wire proportional chamber conceived in 1968 by the CERN physicist Georges Charpak not only opened a new era for particle physics and earned its inventor the 1992 Nobel Prize in Physics but also found important applications in biology, radiology, and nuclear medicine (2, 3).

In 1975, the CERN physicist David Townsend in collaboration with the University of Geneva and the Geneva Cantonal Hospital made important contributions to the reconstruction of PET images and to the development of 3D PET (4–6).

After these individual efforts, CERN witnessed the first collaborative endeavors in medical applications in the 90s. A partnership was established with TERA Foundation (Italy), MedAustron (Austria), and Onkologie 2000 (Czech Republic) to initiate the Proton Ion Medical Machine Study (PIMMS). The PIMMS study is tightly connected to the birth of the ENLIGHT network for hadron therapy in 2002.

In the same years, both the Medipix (7) and Crystal Clear (8) collaborations began exploring possible medical applications of technologies developed for the LHC detectors (hybrid silicon pixel detectors and scintillating crystals, respectively). The Crystal Clear Collaboration developed various PET scanners with variable geometry suitable for both small and large animals. One of them became commercially available to customers worldwide.

Emerging interest in theranostics, i.e., the possibility to perform both imaging and treatment at the same time, has brought radioisotopes for medical use under the spotlight. For the past 50 years, CERN has been hosting ISOLDE, a facility dedicated to the production of a large variety of radioactive ion beams for different experiments in the fields of nuclear and atomic physics, solid-state physics, materials science, and life sciences. Over 1,200 radioisotope beams of more than 70 chemical elements have been made available for fundamental and applied research, including in the medical field. A particular achievement was the demonstration of the efficiency of ^149^Terbium, one of the lightest alpha emitters, for treatment at the level of single cancer cells (9).

### The CERN Medical Applications Office

For several decades, there have been highly successful individual “pockets” of medical technology developments going on at CERN, as well as in the physics communities that have strong formal collaborations with the laboratory. These efforts had considerable success, and the CERN researchers have also been playing key roles in various international multidisciplinary collaborations and networks in specific fields (such as medical imaging, hadron therapy, radioisotopes, data analytics and handling, medical simulations). But a profound shift in the global approach of the laboratory to the whole issue of knowledge transfer to healthcare was needed.

The laboratory shifted a gear in January 2014 by establishing the CERN Medical Applications (CMA) office (10) with the following main goals:

- identify, make available, and foster the applicability of the CERN’s core technologies in accelerators, detectors, simulation, large-scale computing, and data handling that are pertinent to medical applications;
- co-ordinate and structure related activities within the organization;
- catalyze collaborations with external partners, including industry, in a concerted manner.

For the first time in the history of the laboratory, a small amount of “seed” funding and manpower resources for medical applications activities was assigned in the Medium Term (5 year) Plan.

The challenge and aim for the CMA Office is to ensure that state-of-the-art technologies and know-how developed at CERN are used or modified to provide clinical applications that are valuable for the medical community. In order to achieve this goal, the laboratory must prioritize its R&D program for medical applications according to the main concerns and needs of doctors. At the same time, resources for this program should be allocated without compromising particle physics research, which is the core mission of CERN. This process requires the input and guidance of external experts from various disciplines. In keeping with the tried and tested CERN practice, an advisory committee composed of external experts was formed. The committee, called the International Strategy Committee (ISC), comprises specialists from a wide range of medical fields as well as from medical physics. Internally, the Head of the CMA Office is assisted by the CERN Medical Applications Steering Group (CMASG), comprised CERN scientists leading CERN’s projects in the field, as well as of experts from the CERN Knowledge Transfer group and the CERN EU office.

As a first step, the CMA Office identified the key medical physics activities that were already ongoing or were just starting. They included a variety of topics: tools for data handling and data analytics, detectors for medical imaging, radiation dosimetry instruments and techniques, novel accelerators for optimized cancer treatment, facilities for researching new radioisotopes or for biomedical studies, and the vast realm of non-cancer applications.

The ultimate scientific goal of the CMA program is to provide more reliable, more efficient, and more cost-effective treatment options, as well as to ensure early diagnosis of serious illnesses.

## Particle Therapy

The idea of using accelerated beams of protons for cancer treatment was proposed by a visionary physicist and founder of Fermilab, Robert Wilson in 1946 (11). Protons and light ions have unique physical properties. They penetrate a patient with minimal lateral diffusion, depositing most of their energy at the end of their range (in the so-called Bragg peak), effectively sparing healthy tissue on their way to the tumor. In addition, they can be focused into narrow pencil beams allowing a precise radiation dose profile and tumor conformed treatment.

This idea was first tested at the Lawrence Berkeley National Laboratory (LBNL) (12). At the time, the accelerators available were not powerful enough to treat deep-seated tumors. Advancement in accelerator technology coupled with improved medical imaging and computing made proton therapy a viable option for routine medical applications in the 1970s. However, it is only since the 1990s that patients started being treated in clinical settings. The first hospital-based facility to treat patients was at Loma Linda, USA. Since protons are hadrons, proton therapy is also referred to as hadron therapy.

The use of ions increases target conformity on the basis of physics principles, i.e., the dose distribution. In this respect, carbon ions have a smaller lateral penumbra than protons, which may allow a better protection of normal tissue. Also, carbon ions have a higher linear energy transfer (LET) compared to protons and photons, which directly correlates with a higher relative biological effectiveness (RBE). LET of carbon ion beams increases steadily as they pass through the body, reaching a maximum in the Bragg peak region: this property is an obvious therapeutic advantage when treating deep-seated tumors. Carbon ions are also more efficient in hypoxic tumors, which are resistant to both photon and proton radiation. The lower acute or late toxicity of carbon ions compared to protons leads to an enhanced quality of life both during and after cancer treatment (13).

The first dedicated carbon ion facility became operational in 1994 at the Heavy-Ion Medical Accelerator Centre (HIMAC) in Japan (14, 15). In Europe, the first patient was treated with carbon ions at the Gesellschaft für Schwerionenforschung (GSI) laboratory in Darmstadt, Germany, in 1997 (16).

With the growing interest in particle therapy, the first dual ion (protons and carbon ions) clinical facility in Europe, established in Heidelberg, Germany, started treating patients at the end of 2009. This was followed by CNAO in Pavia, Italy, which started treating patients in 2011. The third dual ion center in Europe at MedAustron in Wiener Neustadt, Austria, is expected to start treating patients in 2016.

## Cern and Particle Therapy

CERN has been playing an active role in hadron therapy with the design of a dedicated synchrotron for protons and carbon ions (PIMMS) and the involvement in ENLIGHT. At present, efforts in hadron therapy are focused on establishing a facility to provide ion beams for research.

### The Proton Ion Medical Machine Study

The PIMMS group was formed following an agreement between the MedAustron (Austria) and the TERA Foundation (Italy) to combine their efforts in the design of a cancer therapy synchrotron. The study group was later joined by Onkologie 2000 (Czech Republic). CERN agreed to host this study in its PS Division; the PIMMS team started their work in January 1996, and continued working for a period of 3 years.

Proton Ion Medical Machine Study aimed at producing a synchrotron design optimized for treating cancer patients with protons and carbon ions. The proposed design was detailed in two reports issued in 2000 (17). The PIMMS concept was further enhanced by TERA, and implemented at CNAO and MedAustron. Except the initial design study, CERN has also contributed to the realization of the CNAO and MedAustron treatment centers, in particular with expertise in accelerators and magnets, and with training of personnel. Both projects have been accomplished through networks of national and international collaborations.

### OPEN-Access MEDical Facility

The need for an open-access facility for R&D with ion beams in the context of medical applications was first raised at the ENLIGHT meeting in 2005 in Oropa, Italy. This was reiterated by a wide multidisciplinary scientific community at the 2010 Physics for Health workshop, where CERN was asked to take a lead on this initiative. In order to establish OPENMED (OPEN-Access MEDical Facility), the possibility of modifying the existing CERN low energy ion ring (LEIR) accelerator was evaluated in the open brainstorming session in 2012. Again, the broad positive feedback from the medical and radiobiological communities was received (18).

OPEN-Access MEDical Facility intends to provide suitable ion beams for a multitude of interdisciplinary studies, including radiation biology, nuclear physics models for medicine, detectors and instrumentation for dosimetry, diagnostics, and imaging. OPENMED will complement a few existing or planned beam lines for this kind of multidisciplinary research, providing ample beam time without the constraints of a clinical setting. Ideally, all centers hosting research beam lines should form a pan-European collaborative network that will allocate beam time to researchers in an effective and concerted way.

**Figure 2:**
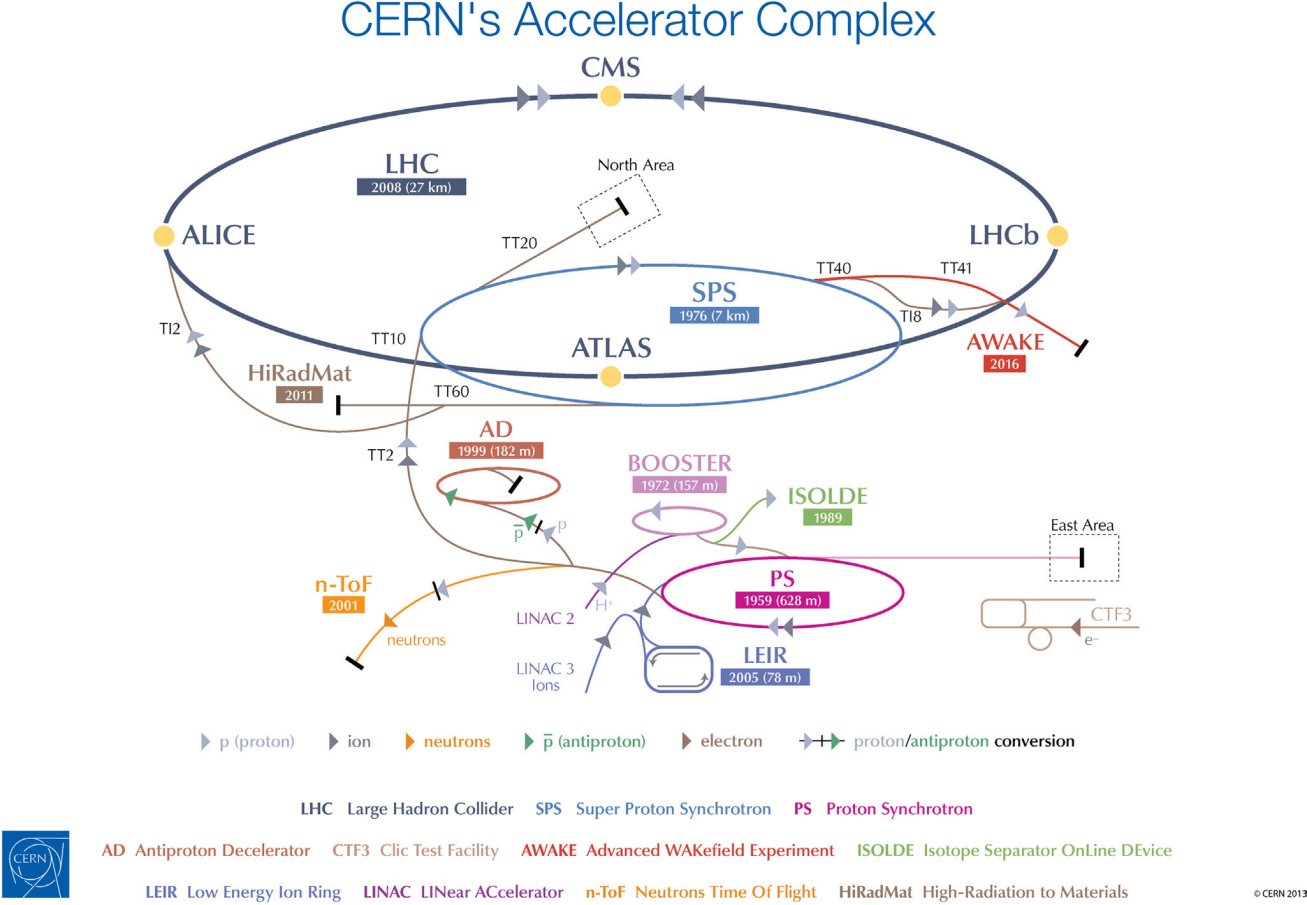
**CERN’s accelerator chain**. The proposed OPENMED facility would use beams from the existing LEIR machine.

The cost of establishing such a facility entirely dedicated to the R&D with ion beams for the medical community will be significantly less at CERN than in a place that lacks the accelerator chain, the expertise to maintain it, and the general infrastructure needed to host the research purposes (see Figure 2). The project entails modifications of the existing LEIR accelerator, which is currently being used for 1 month a year to inject heavy ions into the LHC. The beam energy and size, and its capability of providing up to 9 months of beam time per year, make LEIR an ideal candidate for conversion into OPENMED, which will run without perturbing the scheduled LHC operation (19).

Research at OPENMED will make a significant contribution to the progress of medical physics, biomedical research, medical simulations, and the development of innovative detectors and beam instrumentation.

Studies at OPENMED will ultimately lead to a more safe, optimal, and cost-effective treatment of cancer with radiation. Medical and radiobiological collaborators will be able to investigate the biological impact of different ion beams at various energies on tumor cells and biological materials, and then to optimize radiation therapy for different types of cancer. The research will be carried out on cell cultures and tissues, and it is not foreseen to conduct live animal or human experiments.

In fact, the impressive ion beam radiobiology experiments have been performed over the past 50 years. They were fragmented in time and were performed with different beam qualities and cell systems. All of them need to be systematically reexplored in one single setting, under standardized dosimetry and laboratory conditions, in larger panels of biologically well-characterized human cancer and normal tissue cell systems. The ultimate gain would be a comprehensive model that can individualize therapy, incorporating clinical, biological and physics inputs. Also, it is imperative to develop state-of-the-art instrumentation and methods to bring the performance of hadron therapy to the level of the most advanced photon therapy techniques.

OPEN-Access MEDical Facility will offer ample opportunities for testing novel radiation detectors, medical instrumentation, optimized delivery of the therapeutic beam to patients, diagnostics, and dosimetry. The experimental verification and improvement of biological simulation models will also be possible, along with the studies of complex processes such as nuclear fragmentation.

Experiments at OPENMED will be selected by a panel of experts and carried out within the international collaborations, capitalizing on CERN’s culture of scientific openness, and attracting experts from a variety of fields. OPENMED will become a hub for interdisciplinary exchange, offering R&D opportunities for research in medical radiation biology as well as for the development of a wide portfolio of particle physics techniques, which may be translated into medical applications: detectors, simulation, accelerators, simulation, data handling, and data analytics. These activities would complement other work elsewhere, and contribute to boost the impact of particle physics research on healthcare (20).

## Enlight – The European Network for Light Ion Hadron Therapy

Hadron therapy is the epitome of a multidisciplinary and transnational venture: its full development requires the competences of physicists, physicians, radiobiologists, engineers, and IT experts, as well as collaboration between research and industrial partners. ENLIGHT was established to co-ordinate European efforts in using ion beams for radiation therapy and to catalyze collaboration and co-operation among the different disciplines involved (21). ENLIGHT had its inaugural meeting in February 2002 at CERN and was funded by the European Commission (EC) for its first 3 years (2002–2005).

Despite the end of EC funding in 2005, the following year the network members decided to maintain ENLIGHT alive, with the primary mandate to develop strategies to obtain the necessary funding for hadron therapy research, and to establish and implement common standards and protocols for treating patients. Since then, the co-ordination office has been run at CERN. The current membership exceeds 400 participants from more than 20 countries across Europe.

While the network itself flourishes without the dedicated funding, the R&D activities have been funded primarily through EC projects.

Between 2008 and 2015, four EC projects have been started under the umbrella of ENLIGHT, for a total funding of 24 million Euro: PARTNER, ULICE, ENVISION and ENTERVISION. All these projects are directed toward different aspects of developing, establishing, and optimizing hadron therapy (22).

The Particle Training Network for European Radiotherapy (PARTNER) was a 4-year Marie Curie Training project aimed at educating young biologists, engineers, radio-oncologists, and physicists in the various aspects of hadron therapy. PARTNER provided research and training opportunities for 29 young scientists from a variety of backgrounds and countries between 2008 and 2012, allowing them to actively develop modern techniques for treating cancer in close collaboration with the leading European institutions.

**Figure 3:**
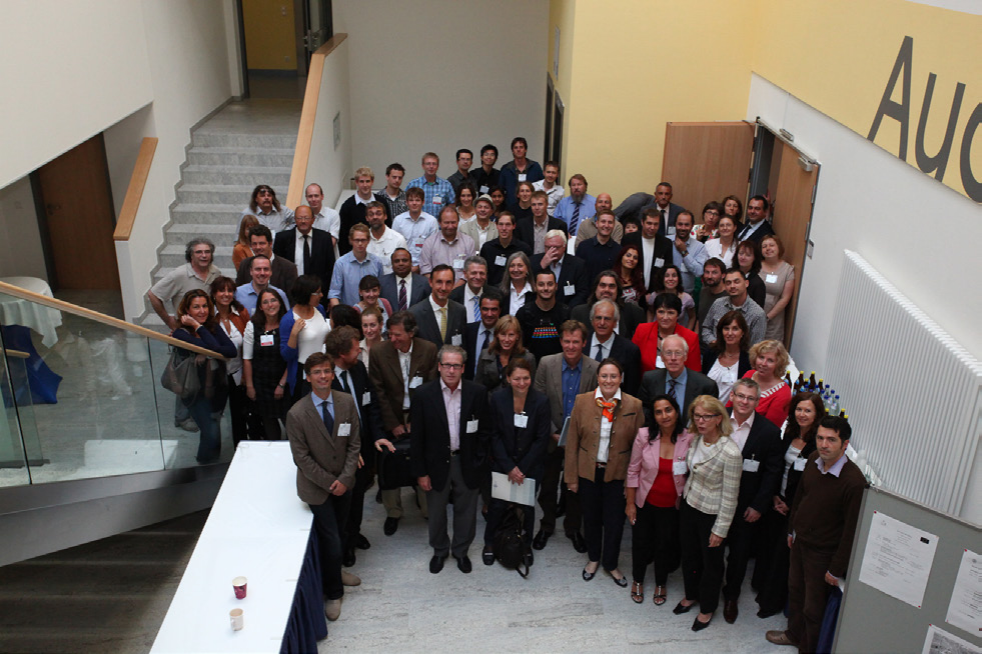
**The ENLIGHT network**. Group picture of the participants to the 2011 ENLIGHT meeting at the Marburg facility, which will start treating patients soon.

The Union of Light Ion Centres in Europe (ULICE) was an infrastructure project which started in 2009 in response to a need for greater access to hadron therapy facilities for clinical and technological research (see Figure 3). The project was built around three pillars: development of instruments and protocols; increasing co-operation between facilities and research communities within the research infrastructure; and transnational access to treatment centers.

A key challenge in particle therapy today is quality assurance during treatment, which needs advanced medical imaging techniques. This issue has been tackled by the ENVISION project, which started in 2010 and covered developments in time-of-flight in-beam PET, in-beam single particle tomography, organ motion monitoring techniques, simulation, and treatment planning. Additionally, ENVISION served as the training platform for ENTERVISION, a Marie-Curie Initial Training Network aimed at educating young researchers in online 3D digital imaging.

### Future Priorities and Challenges

**Figure 4:**
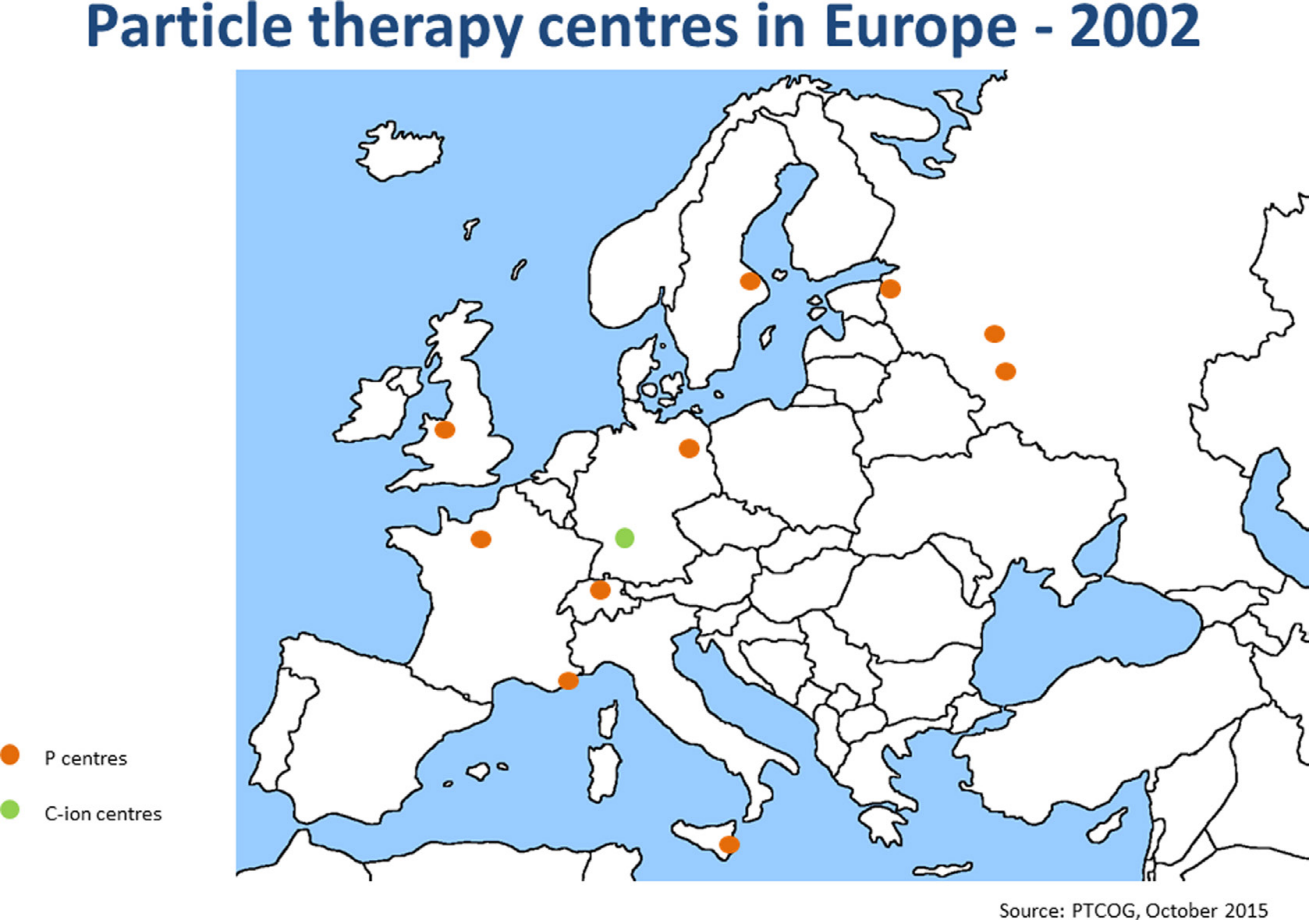
**Hadron therapy centers in Europe in 2002**. This picture shows the distribution and number of hadron therapy centers when ENLIGHT was started.

**Figure 5:**
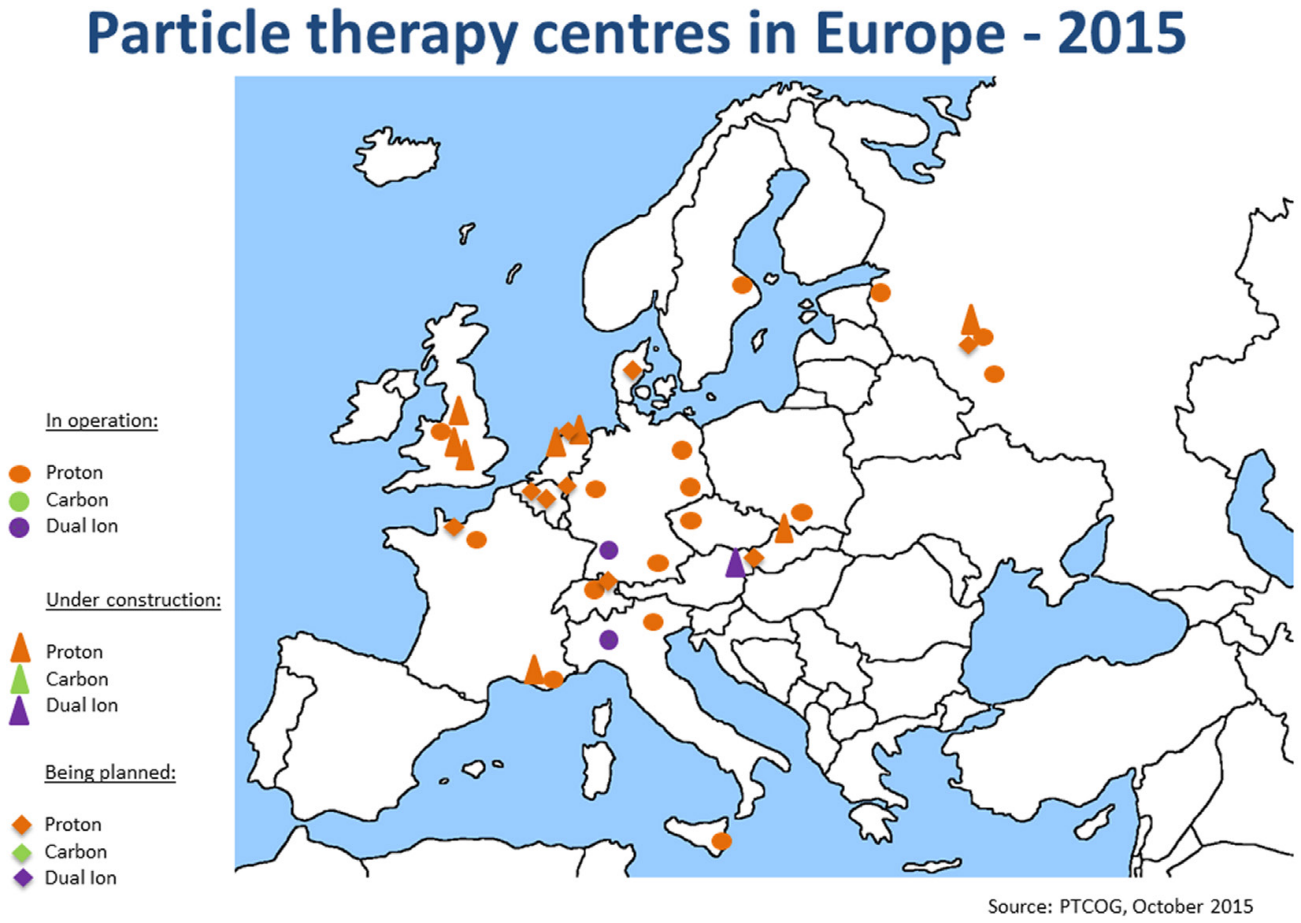
**Hadron therapy centers in Europe in September 2015**. This picture shows the present distribution and number of hadron therapy centers.

Since the annual meeting in summer 2014, the ENLIGHT community has started discussing the future of the network, in terms of both structure and scientific priorities. It is clear that the focus of R&D for hadron therapy has shifted since the birth of ENLIGHT. It is because the number of clinical centers (and especially centers with proton therapy) has dramatically increased (see Figures 4 and 5). Also, while technology developments are still needed in order to ensure increasing accuracy and more cost efficient treatment, further development of proton therapy is now solidly in the hands of industry. The advent of single-room facilities will bring proton therapy, albeit with some restrictions, to smaller hospitals and clinical centers.

From the clinical standpoint, a large number of facilities worldwide would allow the medical community to perform randomized trials to optimize hadron therapy. However, it should be noted that there is a certain reluctance within the hadron therapy community to begin clinical trials from scratch, since a large number of patients has been now treated with both protons and carbon ions, with quite positive results for the main indications. In addition, most of the patients who contact a hadron therapy center are well informed about the treatment, and they expect to be treated with particles.

From the clinical standpoint, the major challenges for ENLIGHT in the coming years will be, on the clinical side, to catalyze collaborative efforts in defining a roadmap for randomized trials (23) and in studying the issue of RBE in details. The efforts concerning technology developments will be continued on quality assurance through imaging and on the design of compact accelerators and gantries for ions heavier than protons. Information technologies will take a central stage, since medical data sharing, data analytics, and decision support systems for patient and treatment selection are key topics.

Providing training will be a major focus in the coming years, as the growing number of facilities requires more and more trained personnel: the aim will be to train professionals who are highly skilled in their specialty but at the same time are familiar with the multidisciplinary aspects of hadron therapy.

## Conclusion

Cross-fertilization between particle physics and medicine continues to be important for improved healthcare. This process needs to be fueled through multidisciplinary exchanges, and geared toward the needs of the medical community. Since 2014, CERN has begun structuring its medical applications activities and has established an international panel of medical and technical experts to assist the laboratory in setting priorities and choosing the future R&D directions.

This paper focuses on the activities related to hadron therapy. CERN has a tight bond with ENLIGHT, since the launch of the network in 2002. ENLIGHT has been very successful in gathering funds for hadron therapy research across Europe and has catalyzed a number of successful projects and collaborations. At present, the ENLIGHT community is establishing a roadmap for the future, taking into account the changes that occurred in the hadron therapy landscape in the past few years.

## Author Contributions

MD – ENLIGHT co-ordinator and CERN Medical Applications deputy, MC – Communication and dissemination officer for CERN Medical Applications, SM – Head of CERN Medical Applications, and SN – Technical contributions to CERN Medical Applications and ENLIGHT activities.

## Conflict of Interest Statement

The authors declare that the research was conducted in the absence of any commercial or financial relationships that could be construed as a potential conflict of interest.
